# Mechanism-based nutritional strategies for performance and recovery in soccer players: a qualitative research synthesis

**DOI:** 10.1080/15502783.2026.2721836

**Published:** 2026-09-01

**Authors:** Amir Hossein Azizi Ammarati, Zohreh Shanazari, Masoud Rahmati

**Affiliations:** a Department of Exercise Physiology, Faculty of Sports Sciences, University of Isfahan, Isfahan, Iran; b Exercise Physiology Department of Physical Education and Sport Sciences, Faculty of Literature and Human Sciences, Lorestan University, Khoramabad, Iran; c Health Service Research and Quality of Life Center (CEReSS), Assistance Publique-Hôpitaux de Marseille, Aix-Marseille Université, Marseille, France

**Keywords:** Melatonin, probiotics, synbiotics, magnesium, athletic performance, exercise recovery, soccer slayers

## Abstract

**Background and objectives:**

Modern soccer, characterized by its intermittent high-intensity nature, requires a combination of explosive power and aerobic endurance. This physiological demand results in substantial metabolic load, oxidative stress, and disruption of recovery processes in players. Multi-target nutritional supplements may therefore provide critical support in maintaining performance and accelerating recovery. This qualitative review synthesizes current evidence regarding the efficacy of melatonin, gut-axis interventions (prebiotics, probiotics, and synbiotics), magnesium, sodium pyruvate, and purple grape-derived products on performance and recovery outcomes in soccer players.

**Methods:**

Twenty-two interventional studies conducted on soccer players were systematically extracted and qualitatively synthesized following PRISMA 2020 reporting guidelines. Methodological quality and risk of bias were assessed using design-specific tools, including the Cochrane Risk of Bias 2 (RoB 2) tool for randomized controlled trials and the ROBINS-I framework for non-randomized intervention studies. Overall certainty of evidence was evaluated using an adapted GRADE framework. Particular attention was directed to study design, dosage and timing protocols, performance and biochemical outcomes, and methodological limitations, with emphasis placed on linking mechanistic evidence with applied performance and recovery outcomes.

**Results:**

Melatonin, administered either nocturnally or prior to exercise, was associated with reductions in oxidative stress markers and upregulation of antioxidant enzymes, contributing to attenuation of short-term performance decline; however, its effects on maximal performance indices were inconsistent. Prebiotic, probiotic, and synbiotic interventions enhanced mucosal immunity and reduced the incidence and duration of upper respiratory tract infections (URTI) as well as delayed onset muscle soreness (DOMS). Certain combined formulations—particularly those co-administered with protein or spirulina—also demonstrated improvements in body composition and muscular strength. Magnesium supplementation alone showed limited and inconsistent benefits in athletes without deficiency, although combined formulations indicated potential improvements in buffering capacity and anaerobic performance. Short-term sodium pyruvate supplementation supported ATP–PCr system resynthesis and enhanced repeated sprint performance. Purple grape-derived products increased serum antioxidant capacity and, in some participants, improved endurance performance.

**Limitations and conclusions:**

Key limitations across studies included small sample sizes, protocol heterogeneity, and a lack of standardized mechanistic biomarkers. Overall, these supplements may support performance and recovery in soccer players; however, larger-scale, field-based randomized controlled trials are required to establish precise, evidence-based operational strategies.

## Introduction

Soccer, as an intermittent team sport, integrates prolonged walking and running with brief bursts of explosive sprinting and repeated anaerobic efforts. Professional players typically cover 10–13 kilometres during a 90–120-minute match [1]. This intermittent movement profile, combined with eccentric contractions and exhaustive training sessions, imposes simultaneous aerobic and anaerobic metabolic stress on skeletal muscle. Such conditions expose muscle tissue to structural damage, increased production of reactive oxygen species (ROS), and disruption of fibre integrity [2,3].

The biological consequences of this physical load include elevated markers of muscle damage such as creatine kinase (CK) and lactate dehydrogenase (LDH), increased lipid peroxidation products such as malondialdehyde (MDA), and systemic inflammatory responses characterised by elevated interleukin-6 (IL-6) and C-reactive protein (CRP). These responses may impair repeated performance capacity and overall match output [2,4]. In addition, congested competition schedules, exposure to artificial lighting, and frequent travel disrupt both sleep quantity and quality. Sleep disturbances further exacerbate oxidative stress, cumulative fatigue, and delayed post-match recovery [5,6].

At the level of immune and mucosal function, intensive training may alter gut microbiota composition and reduce mucosal defence markers such as secretory immunoglobulin A (SIgA). These alterations can increase susceptibility to upper respiratory and gastrointestinal infections while impairing nutrient absorption [7,8].

Given this complex constellation of multi-axis disturbances, reliance solely on training or passive recovery is insufficient for complete physiological restoration. Therefore, targeted nutritional and supplementation strategies are required to concurrently restore redox homoeostasis, mucosal immunity, sleep quality, and energy metabolism [9,10]. Recent systematic reviews indicate that supplements such as melatonin may reduce oxidative and inflammatory markers in athletes; however, evidence regarding consistent and direct improvements in performance parameters remains conflicting [9]. Furthermore, meta-analyses demonstrate that probiotics may reduce the incidence and severity of upper respiratory tract infections (URTI) and attenuate selected inflammatory markers in team-sport athletes, thereby reducing overall training load; nevertheless, optimal strain composition, dosage, and treatment duration remain unclear [11].

The selection of the five supplement categories—melatonin, gut-targeted interventions (pre/pro/synbiotics), magnesium, sodium pyruvate, and purple grape products—was explicitly guided by a multi-axial physiological model of soccer-induced stress. Modern football concurrently disrupts: (i) redox homoeostasis via elevated reactive oxygen species; (ii) neuromuscular function via impaired excitation-contraction coupling; (iii) acid-base balance via repeated anaerobic efforts; and (iv) mucosal immunity via reduced salivary immunoglobulin A. Each supplement was chosen for its distinct mechanistic action on one or more of these axes: melatonin as a chronobiotic antioxidant, probiotics as gut-immune modulators, magnesium as a metabolic cofactor, pyruvate as an intracellular buffer, and purple grape products as sources of vasoactive polyphenols [2,4,7].

Melatonin (*N*-acetyl-5-methoxytryptamine), beyond its central role in circadian rhythm regulation, directly and indirectly scavenges free radicals, induces antioxidant enzyme activity, and exerts significant anti-inflammatory and immunomodulatory effects that may prevent lipid peroxidation and protein damage [12,13]. Emerging clinical trials suggest that melatonin supplementation can reduce oxidative and inflammatory biomarkers while enhancing the activity of antioxidant enzymes such as SOD-1 and GSH-Px. However, findings regarding improvements in maximal or short-term performance parameters remain heterogeneous and appear dependent on dosage, timing of administration, and participant characteristics [12].

Gut microbiota–targeted interventions using probiotics and prebiotics may enhance short-chain fatty acid (SCFA) production, preserve intestinal barrier integrity, and improve mucosal immune markers such as SIgA. These mechanisms may reduce the incidence and severity of URTI and gastrointestinal symptoms while attenuating exercise-induced inflammatory responses. Such effects may directly support recovery capacity and fatigue resistance [7,14,15]. Interventional evidence further indicates that multi-strain probiotics combined with recovery proteins (e.g. casein) may enhance amino acid absorption and improve recovery indices, with some studies reporting reductions in muscle damage markers. However, outcomes related to VO₂max, speed, and strength remain inconsistent and appear highly dependent on strain specificity, dosage, and intervention duration [16].

Magnesium, as a cofactor in hundreds of enzymatic reactions and a central component of the Mg-ATP complex, regulates calcium transport, facilitates actin–myosin cross-bridge formation, and supports mitochondrial metabolism. These mechanisms suggest a potential role in enhancing contraction velocity, energy regeneration, and protein synthesis—key determinants of repeated explosive performance in soccer players [17,18]. Recent systematic reviews indicate that magnesium supplementation may reduce muscle soreness and improve post-exercise recovery, particularly when baseline magnesium status is insufficient or when used in combined formulations [19]. However, clinical evidence suggests that baseline magnesium status represents an important moderator of supplementation response. Ergogenic benefits appear more likely in athletes with insufficient magnesium availability, whereas individuals with adequate baseline status generally demonstrate limited or inconsistent performance improvements. Therefore, differences in baseline magnesium status, in addition to supplement form, dosage, and intervention duration, may partly explain the variability observed across studies [20].

Sodium pyruvate, as a central intermediate in glucose metabolism, may facilitate pyruvate dehydrogenase (PDH) activity, increase oxidative pathway contribution, and act as an intracellular buffer to attenuate lactic acidosis. From a physiological perspective, pyruvate may enhance ATP–PCr system resynthesis and improve repeated sprint tolerance [21,22]. Recent reviews suggest that pyruvate may improve anaerobic performance through acid–base balance optimisation and enhanced oxidative flux; however, reported effect sizes in human studies are small to moderate and require confirmation in larger field-based trials [23]. Existing human data regarding acute or chronic pyruvate supplementation for improving blood pH and post-exercise performance remain heterogeneous and appear dependent on dose, intervention duration, and training protocol [21].

Purple grape products, particularly whole purple grape juice, provide rapidly oxidisable carbohydrates along with bioactive polyphenols including anthocyanins, proanthocyanidins, resveratrol, and related compounds. These bioactives enhance serum antioxidant capacity and may increase nitrate/nitrite bioavailability. This combined energy–antioxidant profile appears suitable for supporting endurance tolerance and tissue protection during intermittent high-intensity training [24,25]. Clinical studies indicate that chronic or strategically dosed purple grape juice consumption may significantly improve time-to-exhaustion and selected endurance parameters in a subset of responders. However, systematic reviews and meta-analyses report increasing heterogeneity and emphasise inter-individual variability—including potential genetic influences—as key determinants of response [26].

Despite growing interest in nutritional supplementation strategies in athletes, there is currently no integrative synthesis linking mechanistic biomarkers with functional performance outcomes specifically in soccer players. Existing reviews have primarily evaluated individual supplements or isolated outcomes, without systematically integrating physiological mechanisms (e.g. oxidative stress, immune function, and acid–base regulation) with soccer-specific performance indicators such as repeated sprint ability, vertical jump performance, and aerobic capacity. This gap limits the ability to translate biochemical responses into practical, mechanism-based nutritional strategies for football players [9,11].

The primary contribution of this qualitative synthesis lies in systematically linking distinct mechanistic pathways—including redox homoeostasis, gut-immune axis modulation, and acid-base buffering—to soccer-specific field performance outcomes. By bridging this gap, the review provides a physiological framework for translating biochemical findings into applied nutritional strategies tailored to the intermittent high-intensity demands of football [2,7,21].

Accordingly, the aim of this article is to integrate the existing mechanistic and clinical evidence regarding melatonin, prebiotics/probiotics, magnesium, purple grape products, and sodium pyruvate; identify current research gaps; and propose applied and investigative priorities for optimising performance and recovery in soccer players [9].

## Methodology

### Study design

This study is a qualitative evidence synthesis conducted in accordance with the PRISMA 2020 reporting guidelines to ensure transparent reporting. This review should not be interpreted as a conventional systematic review with quantitative synthesis. Rather, PRISMA 2020 was applied to ensure transparent literature identification, study selection, and reporting, whereas evidence integration was performed using a qualitative, mechanism-informed narrative synthesis supported by an adapted GRADE framework. Accordingly, the PRISMA flow diagram is presented to document the study selection process, while the final analytical approach remains qualitative rather than quantitative.

In the context of this review, the term “mechanism-informed” (or mechanistic interpretation) refers to the explicit linkage between molecular and biochemical alterations (e.g. changes in serum CK, MDA, SIgA, or blood pH) and corresponding laboratory- or field-based performance outcomes (e.g. RAST total time, VIFT, or Wingate peak power). This approach prioritises studies reporting concurrent biomarker and performance data, thereby providing a physiological basis for interpreting ergogenic and recovery-related effects.

A systematic literature search was performed in the following electronic databases: ISI Web of Science, Scopus, PubMed, and Cochrane Library. The search covered publications from January 2015 to December 2025. The 2015 starting point was selected to reflect a pivotal transition in sports nutrition and exercise physiology research, characterised by a growing emphasis on mechanistic and translational biomarker-based approaches. From this period onward, there was a substantial increase in the use of objective physiological markers such as oxidative stress indices, inflammatory cytokines, immune function markers, and metabolomic profiling in sports performance research. In addition, methodological improvements in study design, assay precision, and performance testing standardisation enhanced the comparability and reliability of findings across studies. Therefore, restricting the search to studies published from 2015 onward ensured methodological consistency and alignment with contemporary evidence-based practices in sports science.

Search terms were structured across three conceptual domains:

**Population:** Football Player, Soccer Player, Athlete.

**Intervention:** Melatonin, Probiotic, Magnesium, Pyruvate, Grape, Polyphenol.

**Outcomes:** Performance, Recovery, Oxidative Stress, URTI, Inflammation.

Boolean operators (AND/OR) were applied to combine the terms. Where available, database filters were applied to restrict results to human studies and clinical trials.

Example PubMed Search String:

(“Soccer”[Mesh] OR “Football”[tiab] OR “Soccer Player”[tiab] OR “Athlete”[tiab]) AND (“Melatonin”[Mesh] OR “Melatonin”[tiab] OR “Probiotics”[Mesh] OR “Probiotic”[tiab] OR “Prebiotics”[tiab] OR “Synbiotics”[tiab] OR “Magnesium”[Mesh] OR “Magnesium”[tiab] OR “Pyruvates”[Mesh] OR “Pyruvate”[tiab] OR “Vitis”[Mesh] OR “Grape”[tiab] OR “Polyphenols”[Mesh] OR “Polyphenol”[tiab]) AND (“Athletic Performance”[Mesh] OR “Performance”[tiab] OR “Recovery”[tiab] OR “Oxidative Stress”[Mesh] OR “Oxidative Stress”[tiab] OR “Respiratory Tract Infections”[Mesh] OR “URTI”[tiab] OR “Inflammation”[Mesh] OR “Inflammation”[tiab]) AND (“2015/01/01”[Date - Publication]: “2025/12/31”[Date - Publication]).

### Study selection process

All retrieved records were screened in two stages: (1) title and abstract screening and (2) full-text eligibility assessment. Two independent reviewers (Z.S. and A.H.A.) conducted both the title/abstract screening and full-text review. Inter-rater agreement between the two reviewers during full-text eligibility assessment was substantial (Cohen’s kappa = 0.89). Any disagreements were resolved through discussion and consultation with a third reviewer (M.R.).

A total of 82 records were screened at the title and abstract level after duplicate removal. Of these, 42 full-text articles were assessed for eligibility. Ultimately, 22 studies met the inclusion criteria and were included in the qualitative synthesis.

Full-text articles were excluded based on predefined eligibility criteria. The most common reasons for exclusion included: (i) absence of a nutritional or supplementation intervention, (ii) lack of soccer-specific or comparable athletic populations, (iii) absence of performance or mechanistic physiological outcomes, and (iv) study designs not aligned with controlled or interventional methodologies (e. g. observational studies, reviews, or case reports). To ensure transparency and reproducibility, detailed exclusion reasons are provided in the Supplementary Material.

### Inclusion criteria

Studies were included if they met the following criteria:

1. Implementation of a nutritional intervention or supplementation protocol
2. Conducted in soccer players or athletic populations with comparable physiological demands. Comparable athletic populations were operationally defined as athletes competing at a professional, semi-professional, collegiate, or elite youth level and exposed to intermittent high-intensity exercise (HIIE) loads similar to soccer, including structured training programs with a weekly training volume of ≥8 hours and regular participation in repeated sprint and high-intensity interval-based activities
  A limited number of studies conducted in non-soccer athletic populations were included only when soccer-specific evidence was unavailable and when these athletes demonstrated comparable physiological characteristics, including repeated high-intensity efforts, anaerobic energy contribution, neuromuscular demands, and recovery requirements similar to those encountered in soccer. These studies were considered as mechanistic supportive evidence and were interpreted separately from direct evidence obtained in soccer players.
3. Because the primary objective of this review was to evaluate mechanistically relevant nutritional strategies for the physiological demands of soccer, a limited number of studies conducted in athletes from sports with comparable metabolic and neuromuscular characteristics were also considered when soccer-specific evidence was unavailable. These studies were included only when they involved trained athletes exposed to repeated high-intensity exercise demands and reported outcomes directly relevant to soccer performance or recovery (e.g. anaerobic power, repeated sprint capacity, oxidative stress, inflammatory markers, or recovery biomarkers). Findings derived from non-soccer populations were interpreted cautiously and were not considered equivalent to direct evidence from football players
4. Reporting at least one performance or biomarker outcome relevant to soccer, including but not limited to: endurance parameters (e.g. VO₂max, Wingate Anaerobic Test [WanT]), repeated sprint performance (e.g. RSA, RAST), muscle damage markers (e.g. CK, LDH), and oxidative stress, inflammatory, or immune markers (e.g. SIgA)
5. Published in peer-reviewed journals between 2015 and 2025

### Exclusion criteria

Studies were excluded if they: -Examined exclusively training, mechanical, or non-nutritional pharmacological interventions; -Included non-athletic or clinically diseased populations unrelated to sport performance; -Did not report mechanistically interpretable physiological or performance-related data; Were review articles, case reports, conference abstracts, or non-peer-reviewed publications. The study selection process is illustrated in the PRISMA flow diagram (Table 1).

**Table 1.** PRISMA flow of study selection.

Stage	Description	*n* (Studies)	Methodological Rationale/Notes
1	Records identified through database searching	70	Systematic search conducted in **PubMed, Scopus, ISI Web of Science, and Cochrane Library** (2015–2025) using predefined keyword combinations.
2	Records identified through other sources	26	Identified via manual reference screening of relevant review articles, targeted searches in key journals in sports nutrition and exercise physiology, and expert domain knowledge (manual reference checking and key journal hand-searching).
3	Total records identified	96	Combined records from electronic databases and supplementary sources.
4	Records after duplicate removal	82	Fourteen duplicate records identified and removed across databases.
5	Records screened (title and abstract)	82	Initial screening based on study population (soccer players) and nutritional/supplementation interventions.
6	Records excluded at screening stage	40	Excluded due to non-soccer populations, non-nutritional interventions (pharmacological or training-only), or absence of performance/recovery-related outcomes.
7	Full-text articles assessed for eligibility	42	Full-text evaluation conducted according to predefined inclusion and exclusion criteria.
8	Full-text articles excluded (with reasons)	20	Reasons included: observational or descriptive design without intervention, lack of quantitative performance or recovery outcomes, low methodological quality, or misalignment with the targeted supplements.
9	Studies included in qualitative synthesis	22	Eligible studies incorporated into the final qualitative mechanistic synthesis.
10	Studies included in quantitative synthesis (meta-analysis)	0	Due to substantial heterogeneity in supplementation protocols, outcome measures, and study designs, meta-analysis was not methodologically appropriate.

Data extraction was conducted using a standardised and pre-piloted data extraction form developed prior to the screening phase. The form was adapted from established reporting frameworks, including the TIDieR checklist, and included predefined domains such as study design, sample size, participant characteristics, supplement dosage and timing, dietary control and placebo conditions, performance-related outcomes, and mechanistic biomarkers. The extraction form was pilot-tested on a subset of included studies and refined accordingly to ensure clarity and completeness. To reduce bias and enhance methodological rigour, all extracted data were independently verified by a second reviewer, and discrepancies were resolved through discussion and consensus. Funding sources and conflict of interest statements for all included studies were also systematically extracted and recorded using the same standardised form to enhance transparency and support assessment of potential sources of study-level bias. The characteristics of the included studies are summarised in Table 2.

**Table 2.** Characteristics of included studies.

Study	Design	Population (*n*, age, level)	Supplement (dose, form, duration)	Training Protocol	Key Outcomes	Methodological Limitations	Contribution to overall certainty of evidence
**Melatonin**							
Farjallah et al., 2022	Double-blind RCT	20 males (18.8 ± 1.3 y), professional (Tunisian First League)	5 mg nightly, oral, 6 days	6-day intensive camp (2 sessions/day), RSA pre/post	↓28% CK; ↓22% MDA (*p* < 0.001); preserved performance	Sleep not assessed; small sample	Moderate
Farjallah et al., 2022	Double-blind crossover RCT	13 males (17.5 ± 0.8 y), professional	6 mg acute, 30 min pre-exercise	Run to exhaustion (100% MAS)	↓37% CK (*p* < 0.001); oxidative protection; no TTE effect (*p* = 0.19)	No power calculation; delayed markers not measured	Low
Czuczejko et al., 2019	Quasi-experimental (no placebo)	47 males (20.9 ± 2.4 y), professional	5 mg nightly, 30 days	Speed-endurance preparation phase	↓MDA; ↓isoprostanes; ↑SOD-1, ↑GSH-Px (*p* < 0.05)	No randomisation/blinding; non-athletic control	Low
Farjallah et al., 2018	Double-blind RCT	20 males (18.9 ± 1.3 y), professional	5 mg nightly, 6 days	6-day training camp; RSA pre/post	Maintained early sprints (*p* < 0.05); ↓MDA; ↑SOD	Blinding details unclear; small sample	Moderate
Farjallah et al., 2020	Quasi-experimental	20 competitive males (age NR)	5 mg nightly, 6 days	6-day camp; RSA pre/post	↓Muscle damage; ↓oxidative stress; improved recovery	Randomisation/blinding unclear; sleep not assessed	Moderate
Farjallah et al., 2022	Double-blind crossover RCT	12 males (17.5 ± 0.8 y), professional	6 mg acute	Run to exhaustion (100% MAS)	↓ASAT, ALAT, GGT (*p* < 0.05); ↓Creatinine (*p* < 0.001); no TTE change	Small sample; no chronic evaluation	Moderate
Ghattassi et al., 2024	Double-blind crossover RCT	12 males (22.9 ± 1.3 y), professional	8 mg single-night	Wingate, squat jump, handgrip	↑9.4% Wingate Ppeak (*p* < 0.001, d = 1.84); ↑30% sleep quality	Limited generalisability (males only)	High
Probiotics							
Badakhshan, 2021	Counterbalanced quasi-exp.	28 young males	Probiotic (910 units/g), 48 h	Hoff & RAST tests	No significant fibrinogen change (*p* = 0.296)	Short duration; small sample	Low
Adikari et al., 2019	Double-blind RCT	19 professionals (UiTM FC)	*Lactobacillus casei* Shirota (1 × 10^9^ CFU), 8 wks	Daily training + matches	↓Cognitive anxiety (*p* = 0.026); ↓Somatic anxiety (*p* = 0.012); ↓Stress (*p* = 0.007)	Mechanisms not assessed	Moderate
Samsamy Pour et al., 2025	Double-blind RCT	40 professionals (Iran)	Probiotic (4.5 × 10^11^ CFU) + Spirulina (2 g), 8 wks	5 sessions/wk strength & plyo	↑Body composition; ↑Agility (*p* = 0.001); ↑Isokinetic strength	No microbiome analysis	Moderate
Jahani Qashlaq, 2016	Quasi-exp.	36 youth males (16–19 y)	Probiotic yoghurt (400 mL/day), 8 wks	HIIT (3 × /wk)	↑SIgA; ↓URTI; ↑Aerobic power	No placebo; no blinding	Low
Mohammadi, 2024	RCT	30 youth professionals	Probiotic (2 × 10^8^ CFU), 2 wks	Wingate & match simulation	↓DOMS; ↓CK (*p* = 0.001); no aerobic change	Short duration; no inflammatory markers	Moderate
Adikari et al., 2020	Double-blind RCT	19 competitive males	*L. casei* Shirota (3 × 10^10^ CFU), 8 wks	Cognitive stress task	↑Theta & delta waves; ↓Reaction time	Limited physiological measures	Moderate
Zhang et al., 2023	Controlled RCT	30 elite students	Synbiotic 2 g/day, 6 wks	75% VO₂max training	↓60% URTI; ↑15.8% SIgA (*p* < 0.01)	No true placebo; males only	Moderate
Sadeghi et al., 2025	Double-blind RCT	44 professionals	Probiotic (4.5 × 10^11^ CFU) + Casein (20 g), 4 wks	RAST; wall-squat	↑Anaerobic power (*p* = 0.003); ↑Lower-body endurance (*p* = 0.001)	No gut microbiome assessment	Moderate
Imanian et al., 2024	Double-blind RCT	44 professionals	Probiotic + Casein, 4 wks	Bruce test	↑TTE; ↑RCP; ↑Time-HHV	No biomarker analysis	Moderate
**Magnesium**							
Chycki et al., 2018	Placebo-controlled RCT	26 elite males	Na/K bicarbonate + Mg citrate, 9 days	RAST (6 × 30 m)	↑2.23% RAST; ↑Resting pH	Combined supplements; small sample	Moderate
Boguth, 2019	RCT	81 NCAA Division-I	Mg (100–200 mg), 6 wks	1RM tests	No significant differences	No blinding; baseline Mg not assessed	Low
Zajac et al., 2020	Placebo-controlled RCT	16 elite males	Mg-creatine chelate (5.5 g/day), 16 wks	Seasonal RAST	↑22% mean power (*p* = 0.005)	No muscle creatine measurement	Moderate
Sodium Pyruvate							
Yang et al., 2022	Double-blind crossover RCT	14 national-level males	0.1 g/kg/day, 7 days	HIIE + RSE cycling	↑13% ATP-PCr resynthesis; ↑8% PPO	No field-based outcomes	Moderate
Purple Grape/Polyphenols							
Niknam et al., 2025	Double-blind crossover RCT	22 U-20 professionals	10 mL/kg acute (4 doses)	30-15 IFT	↑VIFT; ↑VO₂max (*p* < 0.001)	No oxidative biomarkers	Moderate
Atan et al., 2024	Single-blind RCT	36 elite youth	Hardaliye 250 mL/day, 28 days	Standard training + matches	↑TAC; ↓MDA; ↓SBP	No performance measures; single-blind	Low

#### Methodological quality assessment

Methodological quality and risk of bias were assessed according to the design of each included study using appropriate methodological criteria. For randomised controlled trials, risk of bias was evaluated using the Cochrane Risk of Bias 2 (RoB 2) tool, considering domains related to the randomisation process, deviations from intended interventions, missing outcome data, outcome measurement, and selective reporting. For quasi-experimental intervention studies, risk of bias was assessed using the ROBINS-I framework, which considers potential biases arising from confounding, participant selection, classification of interventions, deviations from intended interventions, missing data, outcome measurement, and selection of reported results. This design-specific approach was applied to ensure that methodological limitations were evaluated using criteria appropriate to each study type.

An adapted GRADE-informed approach was applied to evaluate the overall certainty of evidence for each supplement category. The assessment considered five key domains: risk of bias, consistency of findings, directness of evidence, precision of available estimates, and potential publication limitations. Because this review employed a qualitative, mechanism-informed narrative synthesis and substantial heterogeneity existed across interventions, dosages, durations, and outcome measures, certainty ratings were based on the overall coherence, methodological robustness, and reproducibility of findings within each supplement category. Each supplement category was classified as providing high, moderate, low, or very low certainty evidence.

Domain-level risk of bias judgements was directly considered during the certainty assessment process. Specifically, evidence was downgraded when studies within a supplement category showed important methodological limitations, including inadequate randomisation procedures, lack of allocation concealment, absence of blinding or placebo control, high risk of deviations from intended interventions, or incomplete outcome reporting. Conversely, supplement categories supported by studies with lower risk of bias, consistent findings across trials, and adequate methodological rigour were considered to have higher certainty ratings. Thus, the overall certainty rating for each supplement category reflected not only the magnitude and consistency of observed effects but also the underlying methodological reliability of the contributing studies.

Because the number of studies within individual supplement categories was limited and no quantitative meta-analysis was performed, statistical assessment of publication bias (e.g. funnel plot asymmetry or regression-based tests) was not feasible. However, potential publication bias was considered qualitatively during the certainty assessment by evaluating factors such as the limited number of available studies, small sample sizes, predominance of positive findings, and the absence of replication across independent research groups.

### Approach to evidence synthesis

A quantitative meta-analysis was considered during the planning stage; however, it was deemed methodologically inappropriate due to substantial clinical and methodological heterogeneity across the included studies. This heterogeneity was observed across three primary domains: (i) intervention characteristics, including wide variability in supplement categories, dosages, formulations, and acute versus chronic protocols; (ii) outcome measures, encompassing diverse physiological, performance, and biochemical endpoints such as VO₂max, repeated sprint ability (RSA), Wingate peak power, CK, MDA, SIgA, blood pH, and total antioxidant capacity (TAC); and (iii) study design differences, including randomised controlled trials, crossover designs, and quasi-experimental studies. In addition, the limited number of studies within each supplement category precluded meaningful statistical pooling. Therefore, a qualitative, mechanism-informed narrative synthesis was selected as the most appropriate methodological approach. The risk of bias assessment revealed varying levels of methodological concern across the included studies. Detailed risk-of-bias judgements according to study design, using the RoB 2 and ROBINS-I frameworks, are presented in Table 3.

**Table 3.** Risk of bias assessment according to study design using RoB 2 and ROBINS-I frameworks.

Study	Random Sequence Generation	Allocation Concealment	Deviations from Intended Interventions	Missing Outcome Data	Selective Reporting	Outcome Measurement	Overall Judgement
**Melatonin**							
Farjallah et al., 2022	Low (computer-generated code)	Moderate (opaque envelopes)	Low (double-blind)	Low (<5% dropout)	Low (protocol registered)	Moderate (PSQI for sleep)	Some concerns
Farjallah et al., 2022	High (coach-assigned sequence)	High (no concealment)	High (participants identifiable)	Moderate (10% dropout)	High (CK selectively reported)	Low (ELISA assays)	High risk
Czuczejko et al., 2019	High (non-randomised)	High (no concealment)	High (no placebo control)	Low (complete reporting)	Moderate (partial reporting)	Moderate (biochemical assays)	High risk
Farjallah et al., 2018	Low (homogeneous random grouping)	Moderate (identical capsules)	Low (placebo-controlled)	Low (no dropout)	Moderate (selective reporting)	Low (validated performance tests)	Some concerns
Farjallah et al., 2020	Unclear (not reported)	Unclear	Unclear	Low (complete data)	Moderate	Moderate (oxidative biomarkers)	Some concerns
Farjallah et al., 2022	Low (shuffled cards)	Low (identical capsules)	Low (double-blind)	Low (no dropout)	Low (protocol registered)	Low (standardised assays)	Low risk
Ghattassi et al., 2024	Low (statistician-generated code)	Low (opaque packaging)	Moderate (assessor blinding only)	Low (no dropout)	Low (all outcomes reported)	Low (MRS for ATP-PCr)	Low risk
**Probiotics**							
Badakhshan, 2021	Moderate (method not specified)	High (no concealment)	High (no blinding)	Low	Moderate	Low (fibrinogen assay)	High risk
Adikari et al., 2019	Low (random allocation)	Low (identical capsules)	Low (double-blind)	Moderate (1 dropout)	Low (registered protocol)	Low (validated anxiety scales)	Low risk
Samsamy Pour et al., 2025	Low (shuffled cards)	Low	Low (double-blind)	Low	Low	Low (Biodex dynamometer)	Low risk
Jahani Qashlaq, 2016	Moderate (not specified)	High	High (no blinding)	Low	Moderate	Moderate (immune biomarkers)	High risk
Mohammadi, 2024	Low (random allocation)	Moderate (similar capsules)	Moderate (no participant blinding)	Low	Moderate	Low (standardised performance tests)	Some concerns
Adikari et al., 2020	Low (random code generation)	Low (identical packaging)	Low (double-blind)	Low (1 dropout)	Low	Low (EEG & physiological tests)	Low risk
Zhang et al., 2023	Moderate (not detailed)	High (no concealment)	High (dietary variability)	Low	Moderate (IL-10 selective reporting)	Moderate (ELISA)	Some concerns
Sadeghi et al., 2025	Low	Low	Low	Low	Low	Low	Low risk
Imanian et al., 2024	Low	Low	Moderate (assessor-only blinding)	Low	Moderate	Low (Bruce test)	Low risk
**Magnesium**							
Chycki et al., 2018	Low (random allocation)	Moderate (identical capsules)	Low (diet controlled)	Low (<5% dropout)	Low	Low (blood pH analysis)	Low risk
Boguth, 2019	Moderate (within-position allocation)	High	High (no blinding)	Low	Low	Low (strength tests)	High risk
Zajac et al., 2020	Low	Low	Low	High (4 dropouts)	Low	Low (HCO₃^−^ measurement)	Some concerns
**Sodium Pyruvate**							
Yang et al., 2022	Low (1:1 allocation)	Low (identical capsules)	Low (double-blind)	Low	Low (PROSPERO registered)	Low (blood gas analysis)	Low risk
**Purple Grape/Polyphenols**							
Niknam et al., 2025	Low (random allocation)	Low (matched placebo)	Low (double-blind)	Low	Low	Low (metabolic cart VO₂max)	Low risk
Atan et al., 2024	Low (random number generator)	Moderate (identical bottles)	High (participants not blinded)	Low (<5% dropout)	Moderate (secondary outcomes unreported)	Moderate (manual BP measurement)	Some concerns

### Methodological limitations

This review was deliberately designed to focus on interventional human studies conducted in soccer players, with an emphasis on mechanistic depth and translational relevance. While this targeted approach enhanced the physiological interpretability and applied value of the findings, it may have resulted in the exclusion of studies with less direct alignment to performance-oriented mechanistic outcomes.

This review was not prospectively registered in PROSPERO or any other international protocol registry. Although the review was conducted in accordance with PRISMA 2020 reporting guidelines and predefined eligibility criteria, the absence of prospective registration should be acknowledged as a methodological limitation because it may increase the potential for selective reporting bias.

Substantial heterogeneity was observed across key methodological variables, including supplement dosage, formulation, duration of administration (acute vs. chronic), and outcome assessment protocols. This variability precluded the conduct of a quantitative meta-analysis and limited the overall inferential strength of pooled conclusions.

### Population constraints and data quality

The included studies predominantly involved young male soccer players with relatively small sample sizes. This restricts the generalisability of findings to female athletes, different age groups, or other sporting disciplines.

Furthermore, data quality was influenced by several methodological shortcomings in some studies, including incomplete reporting of a priori power calculations, small sample sizes, and the use of quasi-experimental or single-blind designs. Additionally, although the majority of included studies were conducted in soccer players, a small number of studies from comparable athletic populations were included to provide mechanistic insights when soccer-specific evidence was unavailable. However, these findings should be interpreted cautiously because physiological similarity does not completely replace sport-specific evidence.These factors contributed to moderate-to-low certainty ratings for certain outcomes within the GRADE framework and should be considered when interpreting the mechanistic and applied implications of the findings.

All included studies involved exclusively male participants. This was not a predefined restriction imposed by the authors but rather reflected the current availability of interventional literature in soccer-specific nutritional supplementation research. No eligible studies involving female soccer players met the inclusion criteria within the 2015–2025 search period, highlighting a critical gap in the existing evidence base.

## Findings

### Melatonin

**Table:** 

Domain	Summary of Findings	Dose/Protocol	Primary Outcomes	Sources
Mechanism	Direct antioxidant activity; upregulation of superoxide dismutase (SOD) and glutathione peroxidase (GPx); formation of active metabolites	—	Cellular protection; reduction of oxidative damage	[2,27,28]
Chronic nocturnal supplementation	Decreased MDA, AOPP, and isoprostanes; increased antioxidant enzyme activity	5 mg/day during intensive camp periods	Improved recovery; reduced oxidative stress	[2,12,29]
Muscle damage	Reduced CK and LDH during congested training camps	Chronic nighttime dosing	Attenuation of short-term performance decline	[29,30]
Acute pre-exercise ingestion	Reduced post-exercise oxidative stress indices	6 mg, 30–45 min pre-exercise	Biochemical protection without improvement in aerobic performance	[27,31]
Higher nighttime dose	May influence sleep quality and next-day maximal performance (potential effect requiring comfirmation)	8 mg, single nighttime dose	Timing and dose appear critical for ergogenic effect	[28]
Safety	No serious adverse events reported	5–8 mg	Long-term safety data remain limited	[2,28]

Acute vs. Chronic Dosing. It is essential to distinguish between acute pre-exercise ingestion (typically 6 mg, 30 min prior) and chronic nocturnal supplementation (5 mg/day for ≥ 6 days). Acute dosing consistently attenuates post-exercise oxidative stress markers (e.g. MDA, CK) but does not improve time-to-exhaustion or maximal aerobic performance. In contrast, chronic nocturnal regimens mitigate the cumulative decline in repeated sprint performance and reduce muscle damage during intensive training camps, likely through sustained upregulation of antioxidant enzymes (SOD, GPx).

Evidence suggests that melatonin primarily exerts protective antioxidant and recovery-enhancing effects in soccer players, particularly under conditions of intensified training load. Chronic nocturnal administration (5 mg/day) appears beneficial for mitigating oxidative stress and muscle damage markers, whereas acute pre-exercise dosing may confer biochemical protection without directly enhancing endurance performance.

A higher single nighttime dose (8 mg) was associated with improved sleep quality and an observed increase in short-term maximal performance; however, although the increase in Wingate peak power was statistically significant (*p* < 0.001) with a large effect size (d = 1.84), the clinical applicability of this finding remains uncertain because it was obtained from a small, controlled laboratory study and requires confirmation in larger soccer-specific trials.

### Probiotics/Prebiotics/Synbiotics

**Table:** 

Intervention Type	Population/Protocol	Positive Outcomes	Limitations	Sources
Multispecies probiotics	Short-term supplementation	↓ DOMS, ↓ CK	No improvement in VO₂max or aerobic capacity	[32]
*Lactobacillus casei* Shirota	RCT, double-blind, crossover	Improved EEG activity (↑ theta/delta), faster reaction times	Direct physical performance effects not reported	[33,34]
Synbiotic vs prebiotic	Training period	↑ SIgA, ↓ URTI incidence, ↓ inflammatory markers	No change in VO₂max	[7]
Probiotic + Spirulina	Combined supplementation	Improved body composition, agility, strength	Small sample size	[16,35]
Probiotic + Casein	Pre-sleep dosing	↑ Isokinetic strength, ↑ anaerobic capacity	Mechanistic basis unclear	[1]
Summary	—	Strong effects on immunity and recovery	Limited mechanistic data	[1,35]

Strain-Specific EffectsStrain-specific effects. The ergogenic and immunomodulatory effects of probiotics appear to be highly strain-dependent. *Lactobacillus casei Shirota* was associated with improvements in psychological parameters (anxiety, stress) and EEG theta/delta activity, suggesting potential modulation of the gut–brain axis. In contrast, multi-strain formulations (e.g. *Lactobacillus* and *Bifidobacterium* blends) were more consistently associated with reductions in upper respiratory tract infection (URTI) incidence, delayed onset muscle soreness (DOMS), and serum creatine kinase (CK) levels. Therefore, generalisations about “probiotics” as a uniform category are not appropriate.

Immune vs. Direct Performance Outcomes. The evidence strongly supports a beneficial role of probiotics in enhancing mucosal immunity (↑SIgA) and reducing URTI incidence in soccer players. However, direct ergogenic effects on aerobic capacity (VO₂max), speed, or explosive power remain inconsistent and generally non-significant. Observed improvements in anaerobic power or strength were primarily reported when probiotics were co-administered with protein (casein) or spirulina, suggesting that the performance benefit may arise from improved nutrient absorption or synergistic effects rather than a direct action of the probiotic alone.

Probiotic-based interventions consistently support immune function, recovery, and select neuromuscular outcomes in football players, particularly when combined with other ergogenic compounds or timed pre-sleep. Evidence for direct improvements in aerobic performance remains limited, and mechanistic understanding is incomplete.

### Magnesium and related compounds

**Table:** 

Supplement Form	Protocol	Physiological Effects	Performance Outcomes	Sources
Magnesium + bicarbonate	Acute/short-term	↑ blood pH, ↑ HCO₃^−^	Improved RAST performance	[17]
Magnesium alone	100–200 mg/day	Limited physiological changes	No clear effect on strength or jump performance	[36]
Magnesium–Creatine chelate	16-week supplementation	Supports anaerobic metabolism	↑ RAST performance, improved anaerobic indices	[37]
Summary	—	Effects dependent on baseline status	Greater benefit when combined with other compounds	[17,37]

Magnesium supplementation alone appears to have limited and inconsistent effects on performance outcomes in athletes with adequate magnesium status. Although improvements in anaerobic performance have been reported in combination formulations containing magnesium with compounds such as bicarbonate or creatine, these effects cannot be attributed specifically to magnesium because the independent contribution of each component remains unclear. It is methodologically unsound to attribute the ergogenic effect of the magnesium-bicarbonate combination solely to the magnesium component; the improvement is more plausibly explained by the well-established extracellular buffering capacity of bicarbonate. Effects are context-dependent, influenced by baseline magnesium status and training load.

### Sodium pyruvate

**Table:** 

Axis	Details
Physiological Rationale	Enhances acid-base balance, activates PDH, accelerates ATP-PCr resynthesis
Dose & Duration	0.1 g/kg/day for 7 days
Physiological Changes	↑ Blood pH, ↑ HCO₃^−^, ↑ Base Excess (BE)
Performance Outcomes	Improved repeated sprint tolerance and recovery between sprints
Limitations	Small sample size, protocol heterogeneity
Sources	[21]

Sodium pyruvate appears to support short-term anaerobic performance by buffering acidosis and enhancing energy recovery during repeated high-intensity efforts. While the 8% improvement in peak power output (PPO) reached statistical significance (*p* = 0.031), the corresponding effect size was moderate (Cohen's d = 0.86) and derived from a small laboratory-based sample (*n* = 14). The minimal clinically important difference (MCID) for repeated sprint performance in soccer has not been established for this metric; therefore, the practical on-field relevance of this magnitude of change remains to be determined in ecologically valid settings.

### Purple grape & fermented products

**Table:** 

Form	Protocol	Key Effects	Performance Outcomes	Sources
Purple grape juice (acute)	10 mL/kg pre-test	Provides carbohydrates + polyphenols	↑ VIFT, ↑ TTE, ↑ VO₂max	[38]
Purple grape juice	—	—	No effect on explosive strength	[38]
Hardaliye (fermented grape beverage)	250 mL/day, 28 days	↑ Total Antioxidant Capacity (TAC), ↓ MDA, ↓ SBP	Direct performance effects not reported	[39]
**Summary**	—	Effects dependent on dose, form, timing	Individual responses vary	[38,39]

Responder vs. Non-Responder Analysis. Individual responses to purple grape juice supplementation exhibit marked inter-individual variability. In the included trial, 77% of participants demonstrated a meaningful improvement in VIFT (responders), whereas 23% showed no change or a slight decline (non-responders). This heterogeneity is likely influenced by genetic polymorphisms affecting polyphenol metabolism (e.g. SOD3, PPARα) and baseline antioxidant status. Consequently, team-wide blanket supplementation cannot be recommended without prior individual screening.

Interpretation of VO₂peak. Acute purple grape juice ingestion was associated with an increase in VO₂peak measured during the 30–15 Intermittent Fitness Test. This change likely reflects improved exercise tolerance and delayed fatigue rather than a structural adaptation in maximal aerobic capacity, given the acute nature of the intervention.

Polyphenol-rich grape interventions primarily enhance aerobic and oxidative capacity, with antioxidant benefits. Acute supplementation improves high-intensity field performance, whereas chronic fermented forms mainly modulate oxidative stress markers. Individual responses and protocol differences limit generalisability. The overall certainty of evidence for the main outcomes was assessed using the GRADE framework. A summary of the findings and corresponding certainty ratings is presented in Table 4.

**Table 4.** Summary of findings and grade certainty assessment.

Rationale for Downgrading	Number of Studies	Overall GRADE Certainty	Supplement Category
Downgraded one level due to imprecision (small sample sizes)	Moderate	7	Melatonin
Downgraded two levels due to serious risk of bias (lack of blinding in quasi-experimental designs) and indirectness (absence of gut microbiome sequencing).	Low	9	Probiotics/Synbiotics
Downgraded three levels due to extremely serious imprecision (single-study analyses, wide confidence intervals) and inconsistency in formulations.	Very Low	3	Magnesium
Downgraded due to imprecision and indirectness (laboratory-based cycling outcomes).	Very Low	1	Sodium Pyruvate
Downgraded due to imprecision and inconsistency (high inter-individual variability).	Low	2	Purple Grape Products

## Discussion

Football, as an intermittent sport, imposes simultaneous anaerobic and aerobic demands, leading to increased structural muscle damage, reactive oxygen species (ROS) production, and systemic inflammatory responses. Because oxidative stress, inflammation, and muscle damage represent interconnected rather than independent physiological responses to intense exercise, these mechanisms are discussed collectively throughout the manuscript to avoid unnecessary repetition [1,2].

As a chronobiotic and antioxidant compound, melatonin has been shown in intervention studies among football players to reduce oxidative stress markers (e.g. MDA, AOPP, and isoprostanes) and increase protective antioxidant enzymes such as SOD-1 and GSH-Px following chronic nighttime supplementation (e.g. 5 mg). This is accompanied by reductions in CK and LDH, potentially mitigating short-term performance decrements [2,12,29]. Although acute pre-exercise ingestion (6 mg, 30–45 minutes prior) provides biochemical protection, evidence for consistent improvements in endurance or maximal performance is often incomplete or heterogeneous [27,31].

These findings are consistent with broader athletic literature indicating that melatonin’s most reproducible effects involve modulation of oxidative and inflammatory responses, whereas its direct ergogenic effects on sports performance remain uncertain. Therefore, its application in sport should primarily be considered as a recovery- and sleep-supportive strategy rather than a direct performance-enhancing intervention. Therefore, the clinical application of melatonin in sport should focus on antioxidant support and sleep enhancement, with larger randomised controlled trials needed to clarify its role [9].

Concurrently, gut-targeted interventions—including probiotics, prebiotics, and synbiotics—have been shown in studies on football players to increase salivary IgA (SIgA) and reduce the incidence/duration of upper respiratory tract infections (URTI), delayed onset muscle soreness (DOMS), and certain markers of muscle damage. However, their direct effects on VO₂max or power output have been inconsistently observed [7,32,35].

Meta-analytic evidence supports that probiotic supplementation in professional athletes is associated with reduced URTI symptoms and severity, as well as decreased levels of some inflammatory markers. Nonetheless, optimal strain composition, dosage, and duration remain to be clarified [11].

Beyond immune-related benefits, evidence suggests that when probiotics are combined with synergistic nutritional compounds—such as pre-sleep casein or spirulina—positive effects on body composition, isokinetic strength, and anaerobic power may be observed. However, the precise microbe-metabolic mechanisms underlying these synergistic effects require further investigation [16,35]. Nevertheless, where statistically significant improvements were reported, the practical magnitude of these changes remains difficult to determine because studies did not consistently report effect sizes or established thresholds for clinically meaningful performance improvements.

Magnesium, as a biological cofactor in ATP production and neuromuscular transmission, provides a mechanistic rationale for supporting explosive performance and energy recovery. Clinical evidence indicates that baseline magnesium status may act as a key moderator of supplementation efficacy. Benefits appear to be more evident among athletes with inadequate magnesium status, whereas supplementation in magnesium-replete individuals frequently produces heterogeneous or minimal effects. Therefore, assessment of baseline magnesium status should be considered when interpreting study findings and designing future supplementation protocols [17,37].

More recent reviews indicate that magnesium supplementation can reduce muscle soreness and enhance recovery, with performance benefits being more pronounced when magnesium is administered as part of combination formulas (e.g. with bicarbonate or creatine). Accordingly, assessment of baseline magnesium status is recommended prior to supplementation [19].

From a metabolic standpoint, sodium pyruvate may enhance ATP–PCr resynthesis and tolerance during repeated sprints by modulating oxidative pathways and acid–base balance. Short-term intervention studies in football players have reported improvements in ATP–PCr recovery and peak power output (PPO); however, sample sizes were small and field-based evidence remains limited [21]. The sole included pyruvate study utilised a cycle ergometer-based protocol, which, although useful for assessing anaerobic power and metabolic recovery responses, does not fully replicate the eccentric muscle actions, multidirectional changes of movement, and technical demands inherent to field-based soccer. Therefore, extrapolation of these findings to soccer-specific repeated sprint ability and match performance should be interpreted with caution [21].

Recent systematic reviews and evidence summaries indicate that pyruvate has modest but positive effects on peak and mean power during repeated sprint exercises. Nonetheless, the heterogeneity of findings and limited effect sizes highlight the need for confirmation in larger, field-based RCTs [23].

Purple grape-derived products contain both rapidly available carbohydrates and bioactive polyphenols (e.g. anthocyanins, resveratrol, and proanthocyanidins), each of which may contribute to exercise responses through different physiological pathways. The carbohydrate fraction may support exercise performance by providing an immediate energy source, whereas the polyphenol fraction has been associated with enhanced antioxidant capacity and increased nitrate/nitrite bioavailability. However, because the included studies evaluated whole grape-derived products rather than isolated polyphenol preparations or carbohydrate-matched control beverages, the independent contribution of each component cannot be determined with certainty. Therefore, the observed improvements in endurance-related performance outcomes, particularly VIFT, should not be attributed exclusively to the polyphenol fraction, as evidence for improvements in VO₂max remains limited and inconsistent Although the increase in VIFT and VO₂max reached statistical significance (*p* < 0.001), the clinical relevance of these changes should be interpreted cautiously because the intervention was acute, the sample size was limited, and the minimal clinically important difference for soccer-specific intermittent fitness performance remains uncertain [38,39]. Emerging responder-focused analyses further indicate that inter-individual variability is an important determinant of supplementation efficacy. In the included trial, a substantial proportion of participants responded favourably to purple grape juice supplementation, whereas others demonstrated little or no measurable improvement despite receiving the same intervention. These findings reinforce that the ergogenic effects of grape-derived products are not uniform across athletes and highlight the importance of identifying responders and non-responders in future studies. Future randomised controlled trials should incorporate carbohydrate-matched control beverages or isolated polyphenol preparations to better distinguish the independent physiological contributions of carbohydrates and polyphenols to exercise performance and to improve understanding of variability in individual responses and inform more targeted nutritional strategies in football players [38,39].

The integration of these findings suggests that the effectiveness of any supplement depends less on the substance itself and more on its alignment with the target physiological pathway (e.g. oxidation, inflammation, immunity, acid–base balance), the athlete’s baseline status, and the timing of administration. Therefore, future research should explore stratified approaches that consider baseline physiological status and individual variability in response, supported by appropriate mechanistic measures to distinguish potential responders and non-responders.

In practice, this implies that melatonin can be used as an antioxidant and sleep-enhancing strategy during intensive training camps (e.g. 5 mg nightly with monitoring of sleep and biomarkers), probiotics/synbiotics may support daily recovery and reduce the incidence of URTIs, magnesium should be administered based on baseline status and preferably in combination with other compounds, pyruvate may be considered for short-term repeated-sprint strategies, and purple grape products can be selectively used as a natural source of energy and antioxidants.

Overall, the current evidence suggests that these supplements may have potential supportive roles in specific aspects of football performance and recovery; however, the certainty of these conclusions remains limited due to small sample sizes, substantial heterogeneity in dose, formulation, and timing, lack of standardised mechanistic assessments, and limited representation of female athletes. Therefore, current practical applications should be considered preliminary and require confirmation through adequately powered, well-designed, team-based randomised controlled trials incorporating mechanistic and sport-specific outcomes.

### Evidence limitations

A major limitation of the current evidence base is the complete absence of female soccer players in the included studies. All 22 eligible investigations enroled exclusively male participants, resulting in a substantial sex-related evidence gap. This limitation is particularly important because sex-related differences in hormonal regulation, inflammatory responses, oxidative stress adaptation, substrate metabolism, and body composition may influence both physiological responses to supplementation and the magnitude of potential performance effects. Therefore, the current findings cannot be directly extrapolated to female athletes, and dedicated research involving female soccer players across different competitive levels and physiological contexts is urgently required.

The analysis of current evidence highlights significant limitations that challenge both the interpretation and generalisation of findings. These limitations primarily include:

• **Low study quality:** Many investigations suffer from small sample sizes, heterogeneous designs, and short follow-up periods. These limitations substantially increase uncertainty regarding the magnitude and reproducibility of observed effects and reduce the generalisability of findings to broader athletic populations [2,17,35]. Small samples may also increase the likelihood of unstable effect estimates and overinterpretation of positive findings, particularly when replication across independent cohorts is lacking. Furthermore, the predominance of short-term interventions limits conclusions regarding the sustainability, safety, and practical relevance of supplementation strategies across an entire competitive season [2,17,35].
• **Limited ecological validity of performance assessments:** Some performance outcomes were derived from laboratory-based protocols rather than soccer-specific field assessments, limiting ecological validity and restricting the direct translation of these findings to real-world competitive match performance. Although laboratory tests provide valuable mechanistic insights into physiological responses, they do not fully capture the multidirectional movements, technical demands, and intermittent nature of competitive soccer.
• **Limited demographic representation:** Beyond the absence of female participants, many studies predominantly included young male football players, with insufficient representation of different age categories and competitive levels. This restricts the applicability of recommendations across diverse athlete populations and introduces uncertainty in broader generalisations [12,35].
• **Regional publication clustering:** A potential regional publication clustering should also be acknowledged, as a considerable proportion of included studies originated from specific geographic regions, particularly Tunisia and Iran. Although this reflects active research contributions from these settings, it may limit the external validity of the findings due to potential differences in ethnic background, dietary patterns, training environments, environmental conditions (e.g. climate and altitude), competitive level, and sports science support systems. Therefore, replication of findings across diverse populations, countries, and competitive contexts is required before broad generalisation of supplement recommendations to international soccer populations.
• **Lack of long-term intervention data:** Evidence regarding prolonged supplementation (>12 weeks) remains extremely limited across the included studies. Most available trials focused on acute or short-term interventions, which restricts conclusions regarding long-term efficacy, safety, adherence, and practical feasibility during an entire competitive season. This limitation is particularly relevant for supplements intended for chronic use, such as melatonin and probiotics, for which sustained physiological effects and potential risks require further investigation.
• **Publication bias:** Potential publication bias was considered qualitatively as part of the overall evidence assessment. Because the number of studies within each supplement category was limited and no quantitative meta-analysis was performed, statistical approaches such as funnel plot asymmetry or regression-based tests were not applicable. Qualitative indicators suggesting potential publication bias included small sample sizes, limited replication across independent research groups, regional publication clustering, and selective reporting of favourable outcomes. For example, some probiotic and melatonin studies appeared to incompletely report non-significant outcomes (e.g. selected cytokine or sleep-related measures), which may contribute to an overestimation of observed effects and should be considered when interpreting the overall certainty of evidence.

These limitations underscore the importance of conducting future high-quality research with more representative samples, standardised and comprehensive study designs, and longer follow-up periods to achieve a more precise understanding of supplement efficacy and mechanistic actions across diverse athletic populations.

### Overall evidence analysis

An integrated analysis of the evidence indicates that supplement efficacy depends more on the alignment of the supplement with the targeted physiological pathway (e.g. oxidation, inflammation, immunity, acid–base balance), the athlete’s baseline physiological status, and the precise timing of intake, rather than the active compound itself. This pattern may help explain the substantial heterogeneity reported in direct ergogenic effects. Therefore, identifying and differentiating responders from non-responders in future studies is essential to accurately determine supplement efficacy in specific athlete populations.

### Cross-category integration and potential mechanistic complementarity

Although most available studies have investigated individual supplements in isolation, the physiological demands of soccer suggest that a multi-target nutritional strategy may theoretically address a broader range of stressors than single-compound approaches. Different supplement categories appear to influence partially overlapping but distinct biological pathways, including oxidative stress regulation, immune function, inflammation control, energy metabolism, and recovery processes. For example, melatonin and polyphenol-based interventions may primarily target oxidative and inflammatory pathways, whereas probiotics may contribute through modulation of immune function and gut-related responses. Magnesium and sodium pyruvate may influence metabolic processes related to energy availability and cellular recovery.

During congested competitive schedules, when players experience repeated exposure to physical stress, sleep disruption, and increased inflammatory load, theoretically complementary strategies (e.g. combining sleep-supportive interventions such as melatonin with gut-targeted approaches such as probiotics) may provide broader physiological coverage by targeting complementary pathways rather than focusing on a single biological domain. However, current evidence does not allow definitive conclusions regarding additive or synergistic effects of combined supplementation, as controlled trials directly comparing single versus combined interventions in soccer players are currently lacking. Future research should therefore investigate mechanism-based combination strategies using factorial designs and sport-specific performance outcomes.

### Ecological validity and real-world team implementation

Translating findings from controlled supplementation studies into practical soccer environments requires careful consideration of ecological validity. Although the included studies provide important mechanistic insights, real-world team performance is influenced by multiple interacting factors, including training load, match congestion, travel demands, sleep disruption, dietary practices, and individual variability in physiological responses. Therefore, supplementation strategies should not be implemented as isolated interventions but rather integrated within a comprehensive athlete-management framework involving nutrition monitoring, recovery planning, and communication among players, coaches, and support staff.

In applied team settings, the relevance of a supplement may depend on the competitive context and the physiological challenge being addressed. For example, interventions targeting sleep quality, immune resilience, and recovery processes may be particularly relevant during congested fixture periods, whereas strategies aimed at metabolic support or antioxidant capacity may have greater applicability during intensified training phases. However, whether biomarker improvements observed under controlled conditions consistently translate into meaningful changes in match performance remains uncertain. Future applied research should therefore prioritise longitudinal studies conducted within professional team environments, incorporating real-world training loads, dietary monitoring, recovery assessments, and soccer-specific performance outcomes.

### Conclusions and practical recommendations

The overwhelming majority of evidence is derived from small-scale, short-duration studies. Consequently, all practical recommendations presented herein should be viewed as preliminary hypotheses requiring rigorous validation in large, multi-site randomised controlled trials before widespread adoption in elite soccer settings.

Considering the current evidence and applying appropriate caution, the following practical recommendations can be proposed:

• **Melatonin:** Nightly intake of 5 mg during intensive training camps or preparatory periods may support antioxidant defence and mitigate transient performance declines. Monitoring of sleep quality and related biomarkers is recommended [2,30].
• **Probiotics/Synbiotics:** Current evidence suggests that multi-strain probiotics or synbiotics may support immune function and reduce some recovery-related outcomes, particularly the incidence of upper respiratory tract infections (URTI) and delayed onset muscle soreness (DOMS). However, optimal strains, dosages, and intervention durations remain uncertain and require further investigation [7,32].
• **Magnesium:** Combined supplementation with other compounds, such as bicarbonates or creatine, may improve anaerobic performance in elite athletes Standalone magnesium supplementation appears to provide limited benefits in athletes without confirmed deficiency, and further research is required to determine the populations most likely to benefit [17,37].
• **Sodium Pyruvate:** Short-term administration (0.1 g/kg body weight/day for one week) may represent a potential strategy for supporting ATP–PCr recovery and repeated sprint performance; however, current evidence is limited and requires validation in larger soccer-specific trials. Verification in larger cohorts is necessary [21].
• **Purple Grape Juice/Fermented Products:** These sources provide both carbohydrates and polyphenolic compounds, potentially supporting endurance and reducing oxidative stress. However, a targeted approach considering baseline nutritional status and variability in individual responses may be warranted; however, practical responder-screening strategies require further validation.

### Final research recommendations

To advance knowledge on supplement efficacy in athletes and gain a deeper understanding of mechanisms of action and optimal clinical windows, prospective, multifaceted research is essential. Key recommendations include:

1. Advanced Randomised Controlled Trial (RCT) Design:
  ∘ Conduct team-based RCTs involving multidisciplinary expertise (e.g. sports nutritionists, physiologists, sports physicians) for comprehensive coverage of research dimensions.
  ∘ Employ double-blind designs to eliminate bias from participant and investigator awareness of treatment allocation.
  ∘ Utilise power-calculated studies to ensure adequate sample sizes for detecting true effects.
  ∘ Include diverse populations regarding sex and age to enhance generalisability across athlete groups.
2. Standardisation of Supplementation Protocols:
  ∘ Standardise dosage, form (e.g. tablet, capsule, powder), and timing of supplement intake across all study groups. This reduces variance and improves comparability of results.
3. Inclusion of Mechanistic Outcome Measures:
  ∘ Biomarkers: Precisely assess biomarkers related to physiological mechanisms, including:
    ▪ Nitric oxide (NOx) for vascular and blood flow effects.
    ▪ Gut microbiome composition for potential digestive and immune impacts.
    ▪ Anabolic and antioxidant markers (e.g. testosterone, cortisol, GSH, MDA) to evaluate hormonal balance and oxidative stress.
    ▪ Sleep parameters using objective (e.g. actigraphy) and subjective (e.g. questionnaires) tools to assess effects on sleep quality and quantity.

The goal of this comprehensive approach is to directly test causality and identify optimal timing and clinical conditions for maximising supplement efficacy in athletes.

## Supplementary Material

Supplementary Material S_1..docx
