# Supplementary material for: Mechanism-based nutritional strategies for performance and recovery in soccer players: a qualitative research synthesis
Source: J Int Soc Sports Nutr. 2026 Sep 1;23(1):2721836. doi: 10.1080/15502783.2026.2721836 (PMC13537052; doi:10.1080/15502783.2026.2721836)
Supplement: Supplementary file 1 [file RSSN_A_2721836_SM9736.docx]

Supplementary Table S1. Comprehensive Characteristics of the 60 Studies Excluded in the Systematic Review of Mechanism-Based Nutritional Strategies for Performance and Recovery in Soccer Players

| **No.** | **English Title** | **Article Type** | **First Author** | **Year** | **Key Features** |
| --- | --- | --- | --- | --- | --- |
| 1 | Current knowledge about pyruvate supplementation | Review | Robert A. Olek | 2024 | Pyruvate supplementation in athletes; effects on body composition, exercise performance, oxidative stress; emphasizes timing of intake. |
| 2 | Nutritional Habits' Review of Amateur and Professional Football Players | Original Research | Ahmet Göndü | 2019 | 100 players; low nutrition knowledge, inadequate fluid/supplement intake; need for nutrition education. |
| 3 | Use of Magnesium in Exercise and Increased Physical Activity | Review | Darija Vranešić Bender | 2019 | Magnesium role in muscle; athletes at risk of deficiency; Mg citrate improves aerobic/anaerobic performance. |
| 4 | Energy expenditure and dietary intake in professional football players in the Dutch Premier League | Original Research | Naomi Y.J. Brinkmans | 2019 | 41 players; carbohydrate intake low, protein adequate but poorly distributed; need for counseling. |
| 5 | Nutrition and supplementation in football - practical guidelines | Review | Wiktoria Staskiewicz | 2024 | UEFA-based guide; energy, carbs, protein, hydration, supplements (caffeine, creatine, beta-alanine). |
| 6 | To have an intestine with a good health eat food and supplements with Probiotics and Prebiotics | Brief Review | Shameeran Salman Ismael | 2025 | Probiotics/prebiotics for gut health; sources like yogurt, garlic, bananas; reduces inflammation. |
| 7 | Effects of magnesium supplementation on muscle soreness in different type of physical activities | Systematic Review | Maria Grazia Tarsitano | 2024 | 4 studies; Mg (300-500 mg) reduces soreness; 10-20% > RDA recommended. |
| 8 | Effects of melatonin ingestion on physical performance and biochemical responses following exhaustive running exercise | Original Research (RCT) | Omar Hammouda (Farjallah MA) | 2022 | 13 players; 6 mg melatonin; reduced oxidative stress/muscle damage, no performance effect. |
| 9 | Effects of Melatonin on Exercise-Induced Oxidative Stress in Adults with Obesity | Original Research (RCT) | Antonello E. Rigamonti | 2024 | 2 mg melatonin in obese adults; paradoxical pro-oxidant effects; not beneficial. |
| 10 | Effects of Probiotics Supplementation on Risk and Severity of Infections in Athletes | Systematic Review | M. Di Dio | 2022 | 16 studies; probiotics reduce GI and URTI risk/severity (especially Lactobacillus). |
| 11 | Effects of Tai Chi and Baduanjin on muscle mass, muscle function, and activities of daily living in individuals with sarcopenia | Systematic Review & Meta-Analysis | Sen Yang | 2025 | 11 studies (738 participants); improves muscle strength/gait speed, not mass or ADL. |
| 12 | Contribution of Grape Juice to Develop New Isotonic Drinks With Antioxidant Capacity | Review | Yasmina Bendaali | 2022 | Grape juice as natural isotonic base; rich in polyphenols; antioxidant properties. |
| 13 | Effects of Probiotic Supplementation on Exercise and the Underlying Mechanisms | Review | Li Zhang | 2023 | Mechanisms via gut-brain/muscle axes; improves metabolism, immunity, reduces oxidative stress. |
| 14 | Performance variables and nutritional status analysis from Moroccan professional and adolescent football players | Original Research | Mourad Oukheda | 2024 | 253 players; low carb/energy intake; positive correlation between carb/protein and Yo-Yo performance. |
| 15 | How Do Male Football Players Meet Dietary Recommendations? | Systematic Review | Karol Danielik | 2022 | 17 studies; deficiencies in energy, carbs, Ca, Fe, vit D; protein/fat usually adequate. |
| 16 | Role of Magnesium in Skeletal Muscle Health and Neuromuscular Diseases | Scoping Review | Antimo Moretti (Sara Liguori) | 2024 | Mg deficiency linked to muscle diseases; supplementation improves mass, strength, recovery. |
| 17 | Effects of Melatonin on the Body Composition, Physical Performance, and Blood Erythrocyte Indexes of C57Bl/6J Mice | Original Research | S. V. Michurina | 2019 | Animal study; melatonin prevented anemia and lean mass loss, no effect on endurance. |
| 18 | Supplementation of Probiotics and Its Effects on Physically Active Individuals and Athletes | Systematic Review | Gabriella Berwig Möller | 2019 | 24 studies; probiotics reduce respiratory/GI symptoms, limited performance effect. |
| 19 | Probiotics, prebiotics, synbiotics and other microbiome-based innovative therapeutics to mitigate obesity and enhance longevity | Review | Satya Prakash (Boyajian JL) | 2024 | Gut-brain axis in obesity/aging; biotics as novel therapeutics for microbiota modulation. |
| 20 | Can Magnesium Enhance Exercise Performance? | Review | Lijuan Mao (Zhang Y) | 2017 | Animal/human studies; Mg improves performance via glucose increase and lactate reduction. |
| 21 | Dietary Intake, Body Composition, and Nutrition Knowledge of Australian Football and Soccer Players | Original Research | Brooke L. Devlin | 2017 | 66 players; low carb, high protein, low knowledge (57%); knowledge linked to muscle mass. |
| 22 | Nutritional intake, sports nutrition knowledge and risk of Low Energy Availability in female Australian rules football players | Original Research | Dominique Condo (Lohman R) | 2019 | 30 females; low carb (3 g/kg); 30% at risk of LEA; low knowledge (54%). |
| 23 | Pharmacokinetics of magnesium supplements: an in vivo and in vitro approach | Original Research | Wim Derave (L. Blancquaert) | 2019 | 15 Mg supplements; in vitro tests correlate with absorption; Mg citrate > oxide. |
| 24 | Different Approaches to Ergogenic, Pre-, and Probiotic Supplementation in Sports | Mini Review | Joanna Karolkiewicz (Wjacek J) | 2023 | Supplements for aerobic/anaerobic sports; probiotics/prebiotics role in gut health. |
| 25 | The Synergistic Effect of Quince Fruit and Probiotics on Reducing Oxidative Stress and Inflammation and Improving Athletic Performance | Original Research | Martha Rocío Moreno-Jiménez | 2023 | Animal study; quince polyphenols + probiotics increased endurance, reduced inflammation, improved mitochondrial biogenesis. |
| 26 | The Integral Role of Magnesium in Muscle Integrity and Aging | Comprehensive Review | José João Name (Souza ACR) | 2023 | Mg deficiency linked to sarcopenia; supplementation may prevent muscle atrophy. |
| 27 | Impact of Melatonin Supplementation on Sports Performance and Circulating Biomarkers in Highly Trained Athletes | Systematic Review | Diego Fernandez-Lazaro | 2024 | 21 studies; melatonin aids recovery via antioxidant/anti-inflammatory effects; direct performance effect unclear. |
| 28 | The Influence of the Gut Microbiome on Obesity and the Role of Probiotics, Prebiotics, and Synbiotics for Weight Loss | Review | Antoine Aoun | 2020 | Microbiota-obesity link; probiotics/prebiotics help weight loss via SCFAs and inflammation reduction. |
| 29 | Probiotic Supplementation and Respiratory Infection and Immune Function in Athletes | Systematic Review & Meta-Analysis | Karolina Lagowska | 2021 | 14 studies; probiotics reduce URTI severity and IL-6/TNF-α, not infection episodes. |
| 30 | Grape polyphenols supplementation for exercise-induced oxidative stress | Review | Edurne Elejalde | 2020 | Grape supplements reduce oxidative stress; improve antioxidant status and reduce muscle damage. |
| 31 | Nutritional optimization for female elite football players | Topical Review | Maysa V. de Sousa | 2021 | Comprehensive guide for females; focus on energy, iron, calcium, vitamin D, recovery. |
| 32 | Effects of grape juice consumption on muscle fatigue and oxidative stress in judo athletes | Clinical Trial | Cláudia D. Schneider (Goulart MJVC) | 2020 | 14 days grape juice in 20 judo athletes; reduced DNA/lipid damage, increased antioxidant capacity, minor strength improvement. |
| 33 | The effectiveness of home-based physical activity on osteoporosis | Narrative Review | Arianna Murri | 2021 | Home-based exercise (strength/jumping) helps maintain bone density in postmenopausal women. |
| 34 | Walking pace and the risk of stroke | Meta-Analysis | Minghui Quan | 2019 | 7 studies (135,645 participants); fast walking (5.6 km/h) reduces stroke risk by 44%; each 1 km/h increase reduces risk by 13%. |
| 35 | Association between general self-efficacy level and use of dietary supplements in the group of American football players | Original Research | Maria Gacek | 2016 | 100 players; vitamin supplements used more by low self-efficacy; isotonic drinks and whey protein most common. |
| 36 | Evaluation of nutrition intake of football players | Original Research | Heba S. Abdelhaliem | 2018 | 50 adolescent players; high energy/protein/fat, low vit D, Ca, Zn; need nutrition education. |
| 37 | Evaluation of Nutrition and Eating Habits in Children and Adolescents Practicing Football | Original Research | Monika Ameryk | 2016 | 100 boys (11-16); poor habits (low fruit/veg, high fast food/sugar); low healthy diet index. |
| 38 | Nutritional support in professional German football clubs and youth academies—a descriptive study | Original Research (Descriptive Study) | Dolores Reyzis (Hans Braun, Thomas Hauser, Tim Meyer) | 2025 | Survey of 52 German clubs; 83% provide nutrition support; gender disparity; challenges include player resistance and limited resources. |
| 39 | Effects of dietary supplements on athletic performance in elite soccer players: a systematic review | Systematic Review | Rodrigo Abreu | 2023 | 18 studies on elite soccer players; caffeine (3-6 mg/kg) improves jump/sprint; creatine improves sprint/agility; protein improves between-match performance; tart cherry/beetroot aid recovery; sodium bicarbonate + minerals improves anaerobic performance; yohimbine and Resurgex Plus not effective. |
| 40 | Dietary intake of professional Australian football athletes surrounding body composition assessment | Original Research | Sarah Louise Jenner | 2018 | 46 professional players; no one met energy/carb recommendations; knowledge positively linked to energy, protein, fiber intake. |
| 41 | Whole purple grape juice increases nitric oxide production after training session in high level beach handball athletes | Original Research (RCT) | Manoel Miranda Neto | 2020 | 400 ml grape juice; increased nitrite (NO metabolite); antioxidant effect; no change in CK/LDH/CRP/MDA. |
| 42 | Best Practices for Probiotic Research in Athletic and Physically Active Populations: Guidance for Future Randomized Controlled Trials | Review | Alex E. Mohr | 2022 | Framework for probiotic RCTs; strain selection, dosing, study design, outcome measures in athletes. |
| 43 | Pre-season and in-season body composition assessment by bioimpedance in professional football athletes | Original Research | Rand Iblasi | 2025 | 15 players; low energy/carb intake; high protein; growth hormone increased in-season; micronutrient imbalances. |
| 44 | Mechanism of Action and Beneficial Effects of Probiotics in Amateur and Professional Athletes | Review | Yousef Nami | 2024 | Probiotic mechanisms; gut health, immunity, recovery, gut-brain/muscle axes; strain-specific benefits. |
| 45 | Biomarkers of post-match recovery in semi-professional and professional football (soccer) | Review | Íñigo M. Pérez-Castillo | 2023 | Review of recovery biomarkers; CK, LDH, CRP, IL-6, cortisol, oxidative markers; no single gold standard. |
| 46 | The Effects of Melatonin Supplementation on Professional Football Player Performance: A Systematic Review | Systematic Review | Antonio Almendros-Ruiz | 2023 | 8 studies; 5-8 mg melatonin; reduces oxidative stress/inflammation/muscle damage; no direct performance effect. |
| 47 | The Importance of Vitamin D and Magnesium in Athletes | Review | Ligia J. Dominguez | 2025 | Vitamin D & Mg interactions; deficiency common in athletes; essential for bone, muscle, immunity, cardiovascular health. |
| 48 | Polyphenol supplementation boosts aerobic endurance in athletes: systematic review | Systematic Review | Gexin Cao | 2024 | 11 studies; polyphenols improve time-to-fatigue and fat oxidation; limited effect on VO2max; 208 mg/day effective. |
| 49 | Exercise-induced oxidative stress and melatonin supplementation: current evidence | Review | Joanna Kruk | 2021 | Melatonin as potent antioxidant; reduces exercise-induced oxidative stress and inflammation; dose-dependent effects. |
| 50 | An Updated View of the Effect of Probiotic Supplement on Sports Performance: A Detailed Review | Review | Miray Nur Aykut | 2024 | Probiotics reduce GI symptoms, URTI severity, improve recovery; effects on performance still inconclusive. |
| 51 | Sports nutrition applied to soccer: a scientometric analysis | Original Research (Scientometric) | Danilo M. de Oliveira | 2025 | 526 articles (2004-2024); top topics: hydration, energy intake, low energy availability, polyphenols, recovery biomarkers. |
| 52 | Influence of acute melatonin administration on human physical performance: a systematic review | Systematic Review | Vinícius Silva Faria | 2023 | 10 studies; melatonin had no effect on strength/speed/power; improved balance and long-duration exercise in non-athletes. |
| 53 | Lower serum magnesium concentration and higher 24-h urinary magnesium excretion despite higher dietary magnesium intake in athletes | Systematic Review & Meta-Analysis | Haixin Zhang | 2023 | Meta-analysis (14 studies); athletes have lower serum Mg and higher urinary excretion despite higher intake; need higher Mg requirements. |
| 54 | Effectiveness of nutrition education to reduce the risk of sports injury in young soccer athletes | Original Research (Quasi-Experimental) | Yuni Afriani | 2023 | 22 U-16 athletes; education increased knowledge (86.36 to 89.09); focused on antioxidants (vitamin C), hydration, low-glycemic diet; p=0.366 (not significant). |
| 55 | Impact of fish oil supplementation on plasma levels of highly unsaturated fatty acid-containing lipid classes and molecular species in American football athletes | Original Research (RCT) | Veronica Anne Mullins | 2024 | 29 Division 1 players; DHA (2.5g) + EPA (1.0g) ethyl esters for 26 weeks; increased LPC-DHA, FFA-DHA, and phospholipid-DHA/EPA; potential for brain enrichment via BBB. |
| 56 | Lifestyle characteristics in adolescent female football players: data from the Karolinska football Injury Cohort | Original Research (Cohort) | Anne Faltstrom | 2022 | 419 female players (age 14±1); 12% skip breakfast, 8% skip lunch, 16% sleep <8h, 32% stressed, 24% psychological distress; all factors increased at 1-year follow-up. |
| 57 | A Review of Football Players' Sports Nutrition: Protein | Review (Systematic) | Akbar Ghalavand | 2024 | UEFA recommends 1.6-2.2 g/kg/day protein; footballers often have inadequate distribution; protein timing and leucine crucial for MPS; need for nutrition education. |
| 58 | Effects of "Football and Nutrition for Health" program on body composition, physical fitness, eating behaviours, nutritional knowledge, and psychological status among 7 to 10 years school children | Original Research (RCT/Cluster) | Micaela C. Morgado | 2023 | 67 children (7-10y); 12-week intervention; football + nutrition improved BMI z-score, fitness, MVPA, psychosocial health; nutrition group improved fruit/fish intake and knowledge. |
| 59 | Evaluation of the relationship between nutritional status, anthropometric measurements and dietary inflammatory index of professional football players | Original Research (Cross-Sectional) | Emre Batuhan Kenger | 2023 | 21 male professional players; median DII score -3.42 (anti-inflammatory); higher DII correlated with abdominal adiposity (R=0.476, p=0.029); fiber inversely related to abdominal fat. |
| 60 | Nutritional Intake and Training Load of Professional Female Football Players during a Mid-Season Microcycle | Original Research (Repeated-Measures) | C. Leão (Catarina Leão) | 2022 | 14 professional female players (Portuguese League); energy intake (1764 kcal) and CHO below recommendations; training load lower on training days vs. match day; poor correlation between intake and load; mismatch between nutrition periodization and physical demands. |

Supplementary Table S 1. presents the complete list of articles excluded during the title and abstract screening stage (n = 60). Although our PRISMA 2020 flow diagram reports 40 articles excluded at this stage, we provide the full list of 60 articles to ensure maximum transparency and allow readers full access to our screening decisions.

The discrepancy between the number reported in the PRISMA flow diagram (40 articles) and the complete list (60 articles) is explained as follows:

Twenty articles were excluded from the final PRISMA count because they had the least relevance to the primary scope of this review, which specifically focuses on melatonin, gut-axis interventions (prebiotics/probiotics/synbiotics), magnesium, sodium pyruvate, and purple grape-derived products in relation to performance and recovery outcomes in soccer players.

These 20 articles addressed health outcomes unrelated to the scope of the review, such as osteoporosis, stroke, sarcopenia, general inflammation unrelated to exercise, or general child health. Alternatively, they involved non-athletic populations, including sedentary individuals, obese adults, postmenopausal women, or school-aged children, which did not align with the physiological and performance-oriented focus of our review.

All duplicate records (n = 14) were removed before the title and abstract screening stage and were therefore not included in this list, in accordance with the PRISMA 2020 guidelines.

This transparent reporting ensures that our screening process is fully reproducible and that all excluded articles—including those excluded because of limited relevance—are documented and accessible to the reader.

It should be noted that a total of 96 records were identified and accounted for in the screening process. Of these, 14 duplicate records were removed prior to title and abstract screening, 60 articles were excluded during the screening process and are fully documented in Table 1, and 22 articles met the eligibility criteria and were included in the qualitative synthesis. Accordingly, all 96 identified records are fully accounted for (14 duplicates + 60 excluded articles + 22 included articles = 96).
